# Anticancer Effects of Rosmarinus officinalis Leaf Extract on KB Cell Lines

**DOI:** 10.7759/cureus.54031

**Published:** 2024-02-11

**Authors:** V R Pradeep, S Menaka, Vasugi Suresh, Selvaraj Jayaraman

**Affiliations:** 1 Physiology, Saveetha Dental College and Hospitals, Saveetha Institute of Medical and Technical Sciences (SIMATS) Saveetha University, Chennai, IND; 2 Centre of Molecular Medicine and Diagnostics (COMManD) Department of Biochemistry, Saveetha Dental College and Hospitals, Saveetha Institute of Medical and Technical Sciences (SIMATS) Saveetha University, Chennai, IND

**Keywords:** human epithelial carcinoma, anticancer, carboplatin, cisplatin, tumor inducing metalloproteins, real time pcr, mmp-9 gene, mtt assay, kb cell lines, rosmarinus officinalis

## Abstract

Background

The value and use of medicinal plants, including the widespread cultivation of *Rosmarinus officinalis*, have increased rapidly. *R. officinalis*, a medicinal plant native to the Mediterranean, has received attention for its potential therapeutic benefits. This study evaluates *R. officinalis* anticancer activity using human epithelial carcinoma (KB) cell lines derived from nasopharyngeal epidermoid carcinoma. The KB cell line is known for its increased sensitivity to specific chemotherapeutic agents (CA), making it a useful model in cancer research. The impact of *R. officinalis* is assessed using comprehensive analyses of cell viability and gene expression.

Aim

This study aims to evaluate the anti-cancer effects of *R. officinalis* on KB cell lines.

Materials and methods

The *R. officinalis* leaf extract was separated and used to treat KB cell lines. The cell viability of treated KB cells was determined using a 3-(4,5-dimethylthiazol-2-yl)-2,5-diphenyltetrazolium bromide (MTT) assay. Real-time polymerase chain reaction (RT-PCR) was used to analyze the expressions of matrix metalloproteinase (MMP-9) and tumor-inducing metalloproteins (TIMP-1) messenger ribonucleic acid (mRNA) genes. The statistical analysis was performed.

Results

This study analyzes the anticancer properties of *R. officinalis* on KB cell lines. The results show that increasing the concentration of rosemary extract reduces cell viability in malignant cells. Furthermore, the *R. officinalis* effect on the apoptotic signaling system is demonstrated by a decrease in MMP-9 and TIMP-1 mRNA expressions, as observed by RT-PCR analysis.

Conclusion

Patients looking for natural anticancer treatments may benefit from biogenically prepared anticancer drugs. The current research focuses on *R. officinalis* as a potential alternative to chemically synthesized anticancer drugs.

## Introduction

Cancer cells have the characteristic features of a heightened proliferative rate and decreased apoptotic capacity [1]. According to Michael et al., cancer has six characteristics: it continues to produce proliferative signals, it avoids growth inhibitors, it permits replicative immortality, it resists and facilitates cell death, it encourages angiogenesis, and it initiates invasion and metastasis [2]. Cancer progression involves both benign and malignant stages [3]. Genetic predisposition, environmental exposures, and lifestyle choices contribute to its development [4]. Early stages may be symptom-free, necessitating regular screenings and crucial routine examinations, including biopsies for identified abnormalities [5]. Despite accessible clinical evaluation, cancers are sometimes diagnosed at advanced stages. Plant extracts have been explored in cell-line cancer studies for potential therapeutic benefits [6].

Cell lines have the finest track record in cancer research because they reproduce well, proliferate, and multiply quickly. The human epithelial carcinoma (KB) cell line is a valuable tool in cancer research, particularly in anticancer drug development. The KB cell line, which is derived from a human epidermoid carcinoma of the nasopharynx, is widely used due to its susceptibility to a variety of chemotherapy agents. The KB cell line is useful in studying drug resistance mechanisms, providing invaluable knowledge into the problems of dealing with cancer cell resistance to chemotherapy. KB cells are versatile and have been applied to various cancer types, such as cervical cancer, oral cancer, head and neck cancer, lung cancer, breast cancer, ovarian cancer, bladder cancer, pancreatic cancer, colorectal cancer, and prostate cancer [7].

Herbal plants have long been used for therapeutic purposes. They are a natural alternative that can scavenge free radicals and have fewer negative effects. Medicinal herbs, which have significant therapeutic benefits, have been used to treat illnesses. Different medicinal plants provide diverse nutrient-rich benefits. They are packed with many ingredients like fiber, potassium, calcium, phytoconstituents, and diverse nutrients, which add to their powerful therapeutic properties [8]. The shrub Rosmarinus officinalis (R. officinalis) is native to the Mediterranean region. Because of its potent antioxidant qualities, R. officinalis leaves are frequently used in the culinary sector as a spice. Because the rosemary plant contains secondary metabolites like diterpenoids and polyphenols, which have anti-oxidative properties, traditional medicine practices like Ayurveda also use rosemary extracts [9]. Rosemary extract protects against the oral administration of croton oil, which works as a promoter, and 7, 12 dimethylbenzanthracene, which functions as an initiator to cause skin cancer in mice [10]. The spasmolytic activity of rosemary essential oil, which relaxes the muscles and reduces spasms, makes it special [11]. Apart from having anti-oxidative and anti-cancer effects, rosemary extract is also reported to have anti-inflammatory effects. Carnosol is present in the rosemary. It is a high-phenolic diterpene constituent that acts as a potent anti-Parkinson’s agent [12]. Further, rosemary extract is reported to have an antiviral effect against herpes simplex viruses. This is attributed to infusing rosemary extract in an aqueous solution. Essential oil made from R. officinalis leaf has anti-plasmid properties and can carry out antimicrobial activity [6]. The puree of rosemary leaves is used topically for cutaneous infections and atopic dermatitis (eczema). Essential oils made from the leaf extract of R. officinalis contain non-volatile components that have anti-inflammatory activities. Carnosol and carnosic acid are what give rosemary its anti-cancer properties [13]. Carnosic acid is a naturally occurring benzenediol-abietane structure that is primarily present in rosemary and common sage [14]. As a defensive substance, rosmarinic acid guards against illnesses and pests. Rosmarinic acid affects humans in a variety of ways. It is touted as a potent anti-inflammatory and antioxidant substance. From single molecules to the multicellular process that encompasses both invasion and metastasis in animal models, cell lines have been presented in the new domains of cancer biology [15]. The aim of this study is to investigate the potential anti-cancer effects of R. officinalis on KB cell lines.

## Materials and methods

Chemicals

The following supplies were purchased from Gibco, Canada: fetal bovine serum (FBS), trypsin-ethylenediaminetetraacetic acid (EDTA) antibiotics and antimycotics, phosphate-buffered saline, and Dulbecco’s modified eagle’s medium chemicals. The 5,5,6,6-tetrachloro-1,1,3,3-tetraethyl benzimidazole carbocyanine iodide (JC-1) and real-time polymerase chain reaction (RT-PCR) kits were both American products.

Extract preparation

*R. officinalis* extract was created using a Soxhlet device and 70% ethanol. Following filtration, a low-pressure rotary evaporator was used to evaporate it, creating a dense mass that was subsequently maintained at 4 °C.

Purchase and culture of KB cell lines

The KB cell lines (Code: CRL-3596) were bought from the National Centre for Cell Science, Pune, India. In a minimum essential medium (MEM) containing 10% FBS at 37 °C and 5% CO_2_ in the air, KB cells were created.

Cell viability assay

In 96-well plates, the cells were fixed in place overnight at a density of 5 × 105 cells/well. To stimulate the cell culture, different doses of R. officinalis extraction were utilized in triplicate and incubated at 37 °C for 24 hours with 5% humidified CO2. 3-(4,5-dimethylthiazol-2-yl)-2,5-diphenyltetrazolium bromide (MTT) was added to each well after 24 hours of incubation, and the incubation process continued for four hours at 37 °C. Next, 200 μl of dimethyl sulfoxide was used to resuspend the cells and dissolve the formazan that was present in the MTT. To determine optical density, the suspension was analyzed in triplicate at 570 nm in a spectrometer. Every group’s average optical density was measured to determine the value:

$Inhibitory \ rate \ of \ cell \ development \ = \frac{1 - \ OD \ extract \ treated}{OD \ negative \ control} \ast 100$

Gene expression analysis by RT-PCR

Total ribonucleic acid (RNA) was isolated from the samples using Tri Reagent (Sigma, Fukushima, Japan), and 2 µg of RNA from each sample was reverse transcribed using the Superscript III first-strand complementary deoxyribonucleic acid (cDNA) synthesis kit (Invitrogen, Waltham, MA, USA) following the manufacturer's instructions. Real-time polymerase chain reaction (RT-PCR) was performed on the MX3000p PCR machine (Stratagene, Europe) using the MESA Green PCR master mix, which includes SYBR green dye. Eurogentec in the US provided the necessary materials. Melting curve analysis was employed to assess the specificity of the amplified product for each primer combination. Data processing was carried out using the comparative cycle threshold (CT) methodology in CFX Manager Version 2.1 (Bio-Rad, Hercules, CA, USA). The fold change was determined using the 2^(-ΔΔCT) method, as proposed by Schnittger and Livak. 

Statistical analysis

The experiments were performed in triplicate, and the data were shown in ±standard deviation (SD). One-way analysis of variance (ANOVA) was used for statistical analysis, and p < 0.05 was considered to indicate a statistically significant result.

## Results

**Figure 1:**
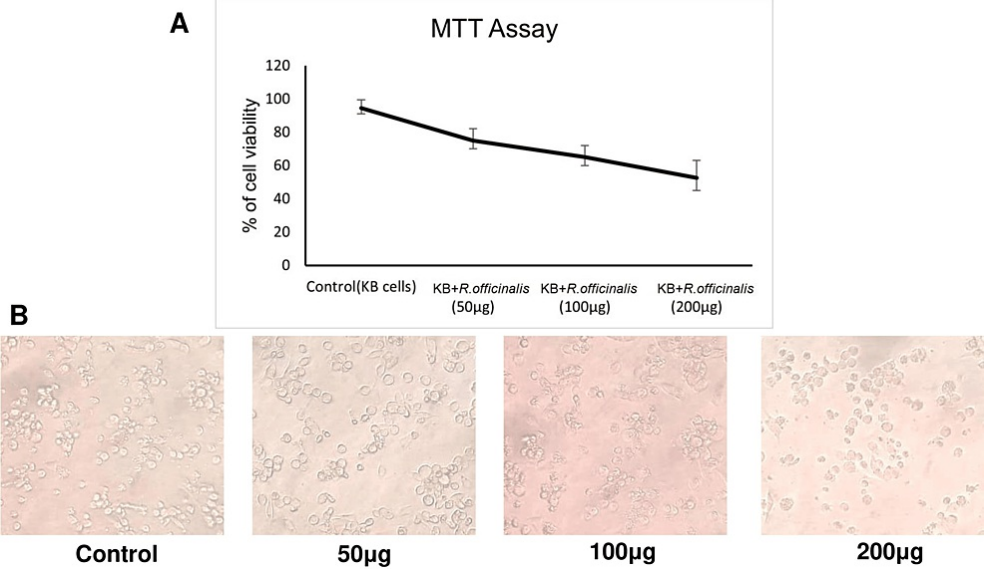
(A) Percentage of cell viability of control and R. officinalis treated KB cell line shown in the MTT assay; (B) morphological changes in control and treated KB cell line in various concentration (50 μg, 100 μg, 200 μg, respectively). MTT: 3-(4,5-dimethylthiazol-2-yl)-2,5-diphenyltetrazolium bromide, KB: human epithelial carcinoma.

In Figure 1, the cell viability of KB cell lines is shown at different concentrations of *R. officinalis* extract. The percentage of cell viability refers to viable cancerous cells. We can find a trend when the extract of *R. officinalis* is added step by step with increasing concentration, and the cell viability of cancerous cells decreases. In the control group (i.e., the untreated group), the percentage of viable cancerous cells was 94.5%, with a standard deviation of 4.95. The minimum inhibitory concentration level was followed, and the percentage of viable cancerous cells reached 52.5% with a standard deviation of 10.61 when the extract was 200 μg/ml. The viability of KB cells decreased with increasing concentrations.

**Figure 2:**
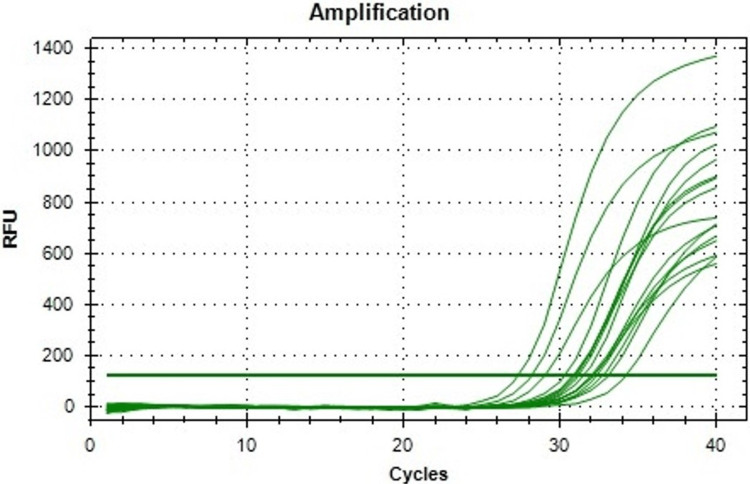
Amplification plot is showing the expression of MMP-9 mRNA in control and R. officinalis extract treated KB cells. RFU: relative fluorescence unit, MMP-9: matrix metallopeptidase 9, KB: human epithelial carcinoma,mRNA: messenger ribonucleic acid.

In Figure 2, matrix metalloproteinase-9 (MMP-9) gene expression was demonstrated in the MMP-9 assay. The MMP-9 gene plays a pivotal role in cancer cells by encoding matrix metalloproteinase-9, an enzyme involved in the breakdown of extracellular matrix components. The MMP-9 gene promotes tumor invasion, metastasis, and angiogenesis in cancer cells. In the control group, the MMP-9 value was 1.0 with no error or deviation. Upon treatment with *R. officinalis*, the values of MMP-9 dropped, and at a concentration of 200 μg/ml, the MMP-9 value reached 0.45 with an error of 0.05.

**Table 1:** Ct values of MMP-9 mRNA expression. KB: human epithelial carcinoma, Ct: cycle threshold, MMP-9: matrix metallopeptidase 9, mRNA: messenger ribonucleic acid.

Control (KB cells)	KB + *R. officinalis* (50 µg)	KB + *R. officinalis* (100 µg)	KB + *R. officinalis* (200 µg)
27	27	30	33
28	26	31	33
29	26	30	34

**Figure 3:**
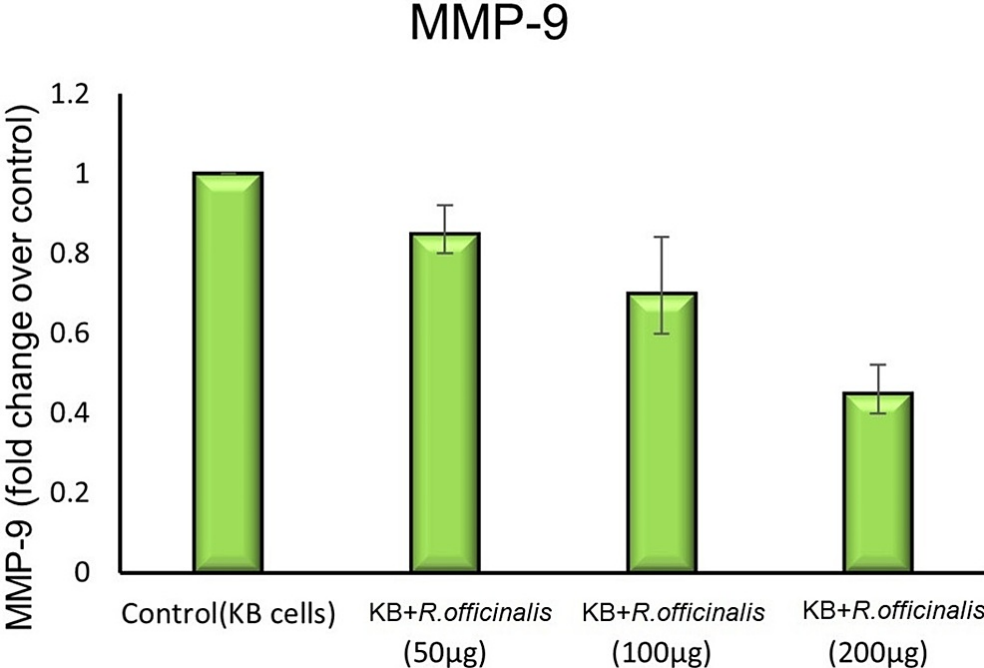
Expression of MMP-9 gene of KB cell control and R. officinalis treated cells were demonstrated in MMP-9 assay. MMP-9: matrix metallopeptidase 9, KB: human epithelial carcinoma.

Figure 3 shows tissue inhibitor of metalloproteinases-1 (TIMP-1) mRNA expression. It is a protein responsible for cell proliferation. Control of this protein will stop the proliferation of cancerous cells. As the concentration of the extract increases, TIMP-1 mRNA expression decreases, resulting in a decrease in the proliferation of cancerous cells. In the control group, the TIMP-1 mRNA value was found to be 1.0 with no error in value. On treatment with R. officinalis, the values of TIMP-1 mRNA dropped, and at a concentration of 200 μg/ml, the TIMP-1 mRNA value reached 0.25 with an error of 0.05.

**Figure 4:**
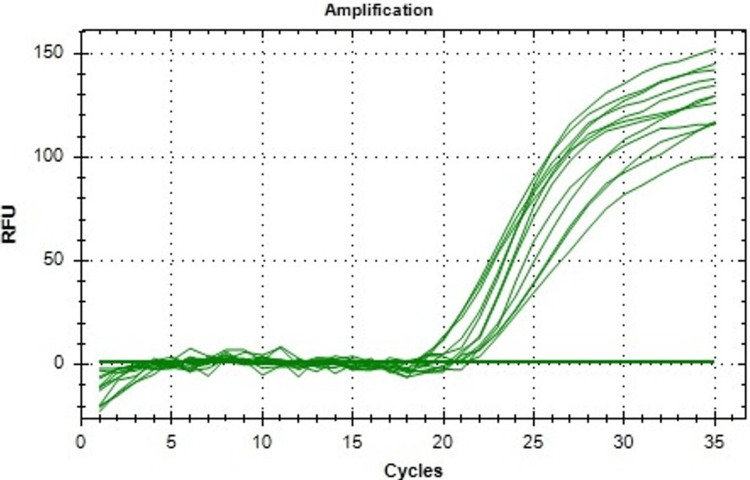
Amplification plot is showing the expression of TIMP-1 mRNA in control and R. officinalis extract treated KB cells. RFU: relative fluorescence unit, KB: human epithelial carcinoma, TIMP-1: tissue inhibitor of metalloproteinases-1, mRNA: messenger ribonucleic acid.

**Table 2:** Ct values of TIMP-1 mRNA expression. KB: human epithelial carcinoma, Ct: cycle threshold, TIMP-1: tissue inhibitor of metalloproteinases-1, mRNA: messenger ribonucleic acid.

Control (KB cells)	KB+*R. officinalis* (50 µg)	KB+*R. officinalis* (100 µg)	KB+*R. officinalis* (200 µg)
18	19	21	24
18	20	21	24
19	20	22	23

**Figure 5:**
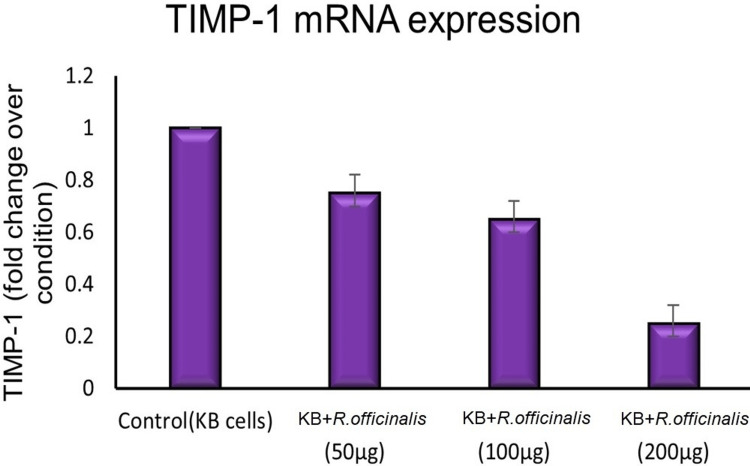
TIMP-1 mRNA expression of KB cell lines treated with different concentrations of R. officinalis. TIMP-1: tissue inhibitor of metalloproteinases-1, mRNA: messenger ribonucleic acid, KB: human epithelial carcinoma.

## Discussion

R. officinalis has been linked to numerous health advantages, including the capacity to treat ailments of the nervous, cardiovascular, gastrointestinal, menstrual, hepatic, and reproductive systems. The anticancer, anti-inflammatory, antioxidant, and attenuating qualities of R. officinalis allow it to treat many diseases that develop in our bodies [16]. Rosemary contains many active components, including carnosic acid, which has potent anti-inflammatory properties. Carnosic acid reduces TNF-α expression and regulates proinflammatory responses. This suggests rosemary may hold promise as a natural remedy for combating inflammation, with potential implications for managing conditions affected by TNF-α and related pathways [17]. R. officinalis was developed and used as a nutraceutical to enhance the anti-cancer effects of already available chemotherapeutic medicines. Numerous projects, including those addressing metabolic diseases, brain dysfunction, and cancer, have demonstrated rosemary’s effectiveness [16,18].

According to the current study, rosemary has excellent potential for use in the treatment of cancer. It can be used as an alternative to already-prescribed medications that have negative effects on human health. According to prior studies on rosemary, a wide range of disorders can be treated or prevented, including cancer, neurological issues, infectious and non-infectious diseases, and cancer. These qualities of rosemary extract have spurred interest in the clinical examination of plant components and prompted researchers to seek advice from early health authorities. In both clinical and preclinical research on the R. officinalis plant, there is an increasing desire for innovative restorative drugs with a specific goal and minimal reactivity [19]. The proliferation of cancer cells can be inhibited by R. officinalis leaf extract. Epithelial tumor cells must penetrate the basement membrane and damage the extracellular matrix (ECM) to spread. Proteases play a vital role in this regard because they can change the components of the ECM, encouraging the diffusion of tumor cells. There is widespread agreement that cancer cells produce more proteolytic enzymes than healthy cells. The urokinase plasminogen activator (uPA) and the MMPs, like MMP-2 and MMP-9, can destroy several ECM components and accelerate the spread and metastasis of cancer [20].

Previous research indicates that cyclooxygenase-2 (COX-2) is upregulated in both human and murine cancer cell lines and that prostaglandin derived from COX can increase MMP delivery and cancer cell motility, promoting metastatic dispersion. Rosemary may potentially slow the spread of cancer by slowing the metastasis process. One-day-later chemotherapeutic agent (CA) treatment prevents the KB cell line from migrating, most likely by reducing the activity of proteases such as uPA and MMPs. Given that it has been demonstrated that CA lowers COX-2 levels in KB cells, these activities may be due to methods involved in the blockage of the COX-2 pathway. Additionally, CA inhibits the adherence of KB cells to surfaces containing fibronectin and type I collagen. Further, the dispersion and pseudopodial extension of CA-pretreated cells have been observed to be inhibited [11]. As an accurate and sensitive measure of cellular metabolic activity, the MTT assay is recommended over other methods, such as adenosine triphosphate (ATP) and the radioactive 3H-thymidine incorporation assay, for determining this end-point. MMP-9, one of the most thoroughly researched MMPs, regulates fibrosis- and inflammation-related structural modification processes in cardiovascular disease. By directly inhibiting ECM proteins, chemokines, and cytokines, MMP-9 regulates tissue rebuilding. TIMP1, also known as TIMP metallopeptidase inhibitor 1, is a glycoprotein with a molecular weight of 28 kDa that performs like a tissue inhibitor of metalloproteinases. This protein is a member of the TIMP protein family [21]. The ECM is degraded by peptidases called MMPs, which the glycoprotein inadvertently inhibits. In addition to these possible anti-apoptotic qualities, the encoded protein can inhibit the majority of known MMPs and boost cell growth in several cell types. The content of the human diet increases the risk of cancer and significantly affects human health. Of the medications now used to treat cancer, 70% are made from natural sources. Rosemary has been identified as a potential anticancer drug due to its antioxidant activity [22].

Numerous studies have been conducted on the antitumor effect of rosemary, which contains more significant quantities of phenolic chemicals. The property of polyphenols is to inhibit anticancer drug resistance. It promotes drug uptake by tumor cells, inhibits drug metabolism by enzymes, and decreases drug efflux. Through these processes, polyphenols increase the sensitivity of cancer cells to anticancer agents [23]. Diterpenes like carnosol and carnosic acid, found in rosemary plants, significantly affect cancer cells. The most well-known cancers that can be treated with these are leukemia, breast cancer, colorectal cancer, hepatocellular carcinoma, and melanoma [24]. Numerous investigations have suggested that direct exposure to carnosic acid can lower the cell potential. It is recommended that the chemicals be used as part of a supplemental anti-tumor strategy [21]. The primary barrier to the successful and efficient treatment of neoplastic disorders is probably chemo-resistance. It has been demonstrated that rosemary and its derivatives can reduce the occurrence of chemo-resistance and improve the impact of chemotherapy [25].

Limitation

It is not practical for humans to consume large amounts of rosemary to achieve significant polyphenol levels that will provide health benefits. Regarding potential future uses of rosemary extract and its polyphenols as anticancer medications, it would make more sense to create a liquid or easily ingested pill containing rosemary extract or its constituents. However, more research is needed to ascertain the effective doses in animals before beginning clinical trials involving humans. Comprehensive studies of animals are also required to ascertain whether long-term drug administration results in toxicity before undertaking clinical trials on humans.

## Conclusions

This study explores the anticancer and cytotoxic properties of R. officinalis when administered to KB cell lines. The treated KB cells were compared to established anticancer drugs such as cisplatin, carboplatin, and 5-fluorouracil. Increasing R. officinalis concentrations caused a gradual decline in KB cell viability, indicating that it has the potential to inhibit the development of malignant cells. The study also analyzes the effect of rosemary plant actions on the apoptotic signaling system, which results in decreased MMP-9 and TIMP-1 mRNA expressions. The anticancer activity of R. officinalis on MMP-9 and TIMP-1 mRNA expressions was confirmed using RT-PCR. These findings indicate R. officinalis's potential therapeutic value in cancer treatment, highlighting its role in key molecular pathways involved in cancer progression.
